# The Book World of Medicine and Science

**Published:** 1897-12-18

**Authors:** 


### The Book World of Medicine and Science.

William Harvey. By D'Arcy Power, F.S.A., F.R.C.S. Masters of Medicine Series. (London: T. Fisher Unwin. 1897. Price 3s. 6d.)

The editors of this attractive series of biographies have done well to give at so early a date the life of William Harvey. It is indeed a life that lends itself to the purposes of the biographer, and Mr. D'Arcy Power has taken such pains in collecting the materials and has shown such skill in telling the story, that a most interesting book has resulted from his labours. We are apt to associate each great man with one discovery or one great deed, and Harvey is associated in all men's minds with the discovery and the demonstration of the circulation of the blood. While, however, the author has quite properly made this great discovery the central fact of the story, he has taken good care not to over-burden his tale with anatomical details. The story is not one of anatomy, but of the life of a man—a man who lived among leaders of thought and action, who travelled much for those days, and who while practising as a physician was the friend and companion of the king; and those were exciting times, times of revolution and civil war, in the stir and bustle of which Harvey had his share. Many curious and interesting details are given regarding the professional and other customs of the times, and it would seem that even in those days questions of hospital abuse and hospital reform were present in men's minds. Thus it appears that the same sort of difficulties in hospital management crop up in different centuries, for even in 1633 we find Harvey recommending to the court of governors of St. Bartholomew's Hospital that steps should be taken to prevent the admission of incurable persons, that persons shall not overstay the time for which they were admitted, "that none lurk here for relief only for slight causes," a point which reformers are still discussing in the present year of grace. The great change which has crept over many professional relationships is strikingly shown by comparing the position occupied by surgeons then and now. The surgeon is such a powerful person nowadays that it is curious to note that in those days the surgeons to a great hospital were debarred from giving "inward physic," even to their own patients, except by the direction of the doctor. In fact, they can hardly be said to have had patients of their own, seeing that even in regard to operations it was ordained that no surgeon should do any great operation, or even "pierce the body," except "with the approbation and by the direction of the doctor." The surgeons did not seem to object to any of these restrictions, but when it was attempted to pass a rule that "every chirurgion shall show and declare unto the doctor . . . what he useth to every external malady," then the surgeons protested, for the arts both of medicine and surgery were then to a large extent secrets, and it did not seem to the surgeons fair that their secrets should be made public while the prescriptions of the physicians were written in a book which was locked and could be read only by the apothecary. The surgeons in those days were evidently kept in curiously complete subjection by the physicians. In his later years Harvey became a great martyr to gout, the paroxysms of which he treated as follows: "He would sit with his legs bare, though it were frost, on the leads of Cockaine House, put them in a pail of water till he was almost dead with cold, then betake himself to his stove, so 'twas gone." Yet he lived to his eightieth year. The book is interesting throughout, not only as the life of one of our greatest masters of medicine, but as giving much insight into the manners of the times. To recognise how far Harvey was in advance of those among whom he lived it is worth recording that in 1634, when he was fifty-six years old, he had to superintend the examination of some women who were stated to belong to "a huge pack of witches," concerning whom Sir William Pelham wrote to Lord Conway as follows: "It is suspected that they had a hand in raising the great storm wherein his Majesty was in so great danger at sea in Scotland." The picture of Harvey's life and investigations is made all the more striking by being set in a frame which shows the surroundings amid which he lived.

On Food and Exercise. By A. Rabagliati, M.D., &c. (London: Baillière, Tindall, and Cox. 1897. 8vo. Pp. 220. Price 5s. net.)

This volume is an expansion of an essay which appeared some months ago in *The Scalpel*. Its purpose is, the author states, to elucidate the proposition that there are three chief predisposing causes of disease—to wit, improper relations between the human organism and air, food, and exercises. Dr. Rabagliati thinks that, in considering the etiology of disease, heredity may be disregarded, and that "exciting causes" are relatively unimportant. The main thesis, however, developed in this book, is that such diverse diseases as herpes, labialis, bronchitis, rheumatism, caruncle (*sic*), apoplexy, and carcinoma are all manifestations of a hypothetical blood state, named by the author "Triphthæmia Carbonifera." This blood state is considered due to excessive ingestion of "carboniferous" articles of diet. The facts offered as evidence of so remarkable an hypothesis are painfully inadequate; and the subordinate theories put forward are certainly original. Bronchitis is declared to be clearly due to excess of starchy or sugary foods; and bronchitic asthma, it is suggested, may be caused by an œdematous condition of the bronchial mucosa due to a growth in it of saccharomyces. The saccharomyces is spoken of as "a bacillus," and we are told that, when in good health, "we oxidise germs off, and suffer nothing." Few physicians will, we hope, accept the dictum that "typhoid fever is generally due to bad air, and sometimes to bad food"; or the suggestion that herpes labialis and pruritus ani are the "same disease in different situations." The most remarkable assertions made by Dr. Rabagliati are those referring to cancer. Cancer is "a dyscrasia or culmination of mal-nutrition in tissue" (p. 26); it is "brought on by an excess consumption of saccharine and starchy foods" (p. 136); and the suggestion that "starvation offers the only hope for relief or possible cure" (p. 182) is spoken of with approval. The evidence offered in support of the "carboniferous" theory of cancer is little more than the statement that Mrs. S., who died of cancer, ate bread four times a day, and Miss F., another sufferer, was fond of cake, and sugar in her tea. No doubt a very large amount of dyspepsia, especially among women of the lower classes, is due to the troubles of carbohydrate digestion; but to fancy that such dyspepsia can ever set up "malignant ulceration of the mouth of the womb" (p. 140) is a use of the imagination other than scientific.

